# Fair Play

**Published:** 1890-08-02

**Authors:** 


### The Hospital

A Weekly Journal of

Science, Medicine, Nursing, and Philanthropy.

*For Hospitals, Asylums, Medical Practitioners, Students, Nurses, Families, Parishes, Congregations, and the Charitable Public.*

Vol. VIII.—No. 201.] [August 2, 1890.

### FAIR PLAY.

The London Hospital has been somewhat briskly handled by the critical investigators of the Lords' Commission. The nursing department, the salaries of officials, the method of keeping accounts, and, practically, every other vital question of hospital management has been probed to the bottom. The London, or, at any rate, some of its officials, have enemies. That is clear. Enemies are generally unpleasant, but not always unprofitable. They can hunt up scandals, and expand short tales into volumes; they can magnify faults, and make the worse appear the better reason; they can impute motives which never existed, and give to circumstances that are perfectly innocent a sinister and malevolent aspect. All this is quite easily understood by those who have once or twice faced cross-examination in a law court. The arts of the cross-examining counsel, which are supposed to be his distinguishing specialty and crowning glory as a lawyer, are, in fact, not a lawyer's specialty at all, but are the natural endowment of all sorts of people who cherish spite, and hate their opponents.

It was foreseen that the Commission would furnish an arena for the fighting out of hospital quarrels. That is not particularly to be regretted. For, notwithstanding the labours and the teachings of the Peace Society, it seems to be quite certain that the wager of battle is sometimes the only way of settling the rights and wrongs of a question. The London Hospital, we have no doubt, is glad of the opportunity of vindicating its character, and making known its worth. It has not sought conflict. It probably appreciates as much as any private person the advice of Polonius: "Beware of entrance to a quarrel." It may be taken for granted, however, that the second part of the aged noble's counsel on this point will also be remembered, and will give steadiness and purpose to the behaviour of the managers in the conduct of their case. Being in the quarrel they will so bear it that the opposed may beware of them. There is no stopping short now of a full investigation of every detail of daily life and management in connection with the Hospital. If the adversaries should retire from the contest; or if the Commission should permit the keen edge of its inquiry to be blunted, the managers themselves owe it not only to their reputation, but even to the existence of their institution, to make full display and exposure of every fact in their present and recent history.

But the throwing of the London Hospital into the seven-times-heated furnace of critical investigation has established a precedent which cannot be overlooked. If the London, the managers will say, why not every other general hospital in London; and if every general hospital, why not every special hospital as well! The force of this argument cannot be resisted. An injury of the gravest character would be inflicted upon the most useful of all the metropolitan charities, if it alone had the peculiar distinction of a detailed inquiry into every circumstance, great and small, of its work and management. One very important result, therefore, of the action of those who have assailed the London Hospital will be that the investigations of the Committee will be marked by a thoroughness and a breadth which will make them conspicuous in the history of Royal Commissions. Every hospital in London must now prepare itself for the searching fires of criticism. Those persons in all parts of the metropolis who know, or think they know, anything against a particular institution will render a public service by offering themselves as witnesses before the Lords' Commission. The spirit of fair play, which every Englishman regards as one of his most honourable and distinguishing endowments, demands that the course of thoroughness having once been entered upon, shall be pursued to the very end.

It has frequently happened that there has been the greatest possible contrast between the searching character of an inquiry by Royal Commission and the futile feebleness of its final recommendations. That has been mostly due to the fact that a great variety of public and private "interests" have been involved in the inquiry; and Royal Commissioners for the most part have a profound veneration for established interests, and an almost ludicrous dread of harming even the very outermost fringes of them. But in the case of hospitals there are no interests of any kind which the Lords need fear to deal with. Hospital subscribers are quite a different class of persons from shareholders in companies, owners of small properties, manufacturers whose business is carried on in unhealthy factories, brewers, publicans, and the like. There is this essential difference between subscribers to charities and persons who have delicate "interests"—that the former make no profit out of their charitable enterprises, but, on the contrary, have every inducement to keep expenditure down at a minimum. No more than the subscribers have the patients any interest in maintaining the *status quo*, if the *status quo* is found on investigation to be characterised by abuses and circumstances that are publicly injurious. The highest well-being of the patients as well as the deepest satisfaction of the subscribers will be secured and conserved by that kind of hospital management with which no real fault can be found. The only class of persons whom the Commission may possibly not desire to touch, or to seriously interfere with, are the paid officials of the hospitals. But we are convinced, as the result of a wide knowledge of individuals and facts, that those officials have no more reason to dread inquiry, or to resist change, than have subscribers and patients. In making its recommendations, therefore, the Commission will do well to avoid speaking with "bated breath and whispering humbleness." Whatever the Lords in their sober wisdom may think to be the best and most desirable that let them recommend with clear precision and unflinching firmness. If they do this all the parties concerned will regard their work with respect and gratitude.

The time has not arrived for the public to sit in judgment upon the judges. But there are abundant signs that the interest in the inquiry is not only deepening week by week, but is spreading to a class of people who have hitherto known little of Hospitals except their names. The whole question of voluntary charity is in fact on its trial. Already the witnesses are beginning to speak of State aid. That is a part of the subject fraught with wide-spreading consequences. It was bound to come up sooner or later in the course of the inquiry; and it is well that it has been brought to the front sooner rather than later. It is one of the most important of all the questions to which the Commission can have its attention directed. Every man who holds strong opinions on the subject, if he have any right to hold them, should seek to press those opinions upon the public attention through the Commission. It is probable that hospital affairs, which have been so long acted upon by a variety of solvents, and which under the influence of the Lords' investigations, are rapidly becoming quite fluid, will crystalise again when the inquiry is over, and will retain their new forms for many years to come. It is eminently desirable, therefore, that nothing shall be left unsaid or undone which can in any way contribute towards the improvement and perfection of those institutions which are among the very noblest of which this country can boast.

The Commission will not be able to complete its work before the Parliamentary recess. Those interested in the questions raised will have time to reconsider their opinions, and to give form and definitiveness to any new judgments they may have arrived at. It is necessary to insist that the London Hospital shall not be made a marked institution by the action of the Commission. Its work is such that it cannot be impaired in the least without consequences to the East-end population which will be nothing short of calamitous. All the other general hospitals of the metropolis must recognise that it is their duty to stand by the London, and to identify themselves with it. This they will best do by voluntarily demanding to be subjected to as broad and searching an investigation as it has experienced. The period of inactivity which is immediately before the Commission will be well spent by the friends of all the voluntary hospitals of the metropolis in preparing for and insisting upon an investigation exactly the same in kind as that which has been conducted in the case of the London Hospital. The same measure must in fairness be meted out to all as has been meted out to it.

